# IL-33/ST2L Signaling Provides Neuroprotection Through Inhibiting Autophagy, Endoplasmic Reticulum Stress, and Apoptosis in a Mouse Model of Traumatic Brain Injury

**DOI:** 10.3389/fncel.2018.00095

**Published:** 2018-04-25

**Authors:** Yuan Gao, Ming-yang Zhang, Tao Wang, Yan-yan Fan, Lin-sheng Yu, Guang-hua Ye, Zu-feng Wang, Cheng Gao, Hao-chen Wang, Cheng-liang Luo, Lu-yang Tao

**Affiliations:** ^1^Department of Forensic Science, Medical School of Soochow University, Suzhou, China; ^2^Department of Forensic Science, Wenzhou Medical University, Wenzhou, China

**Keywords:** autophagy, endoplasmic reticulum (ER) stress, IL-33-ST2L signaling, neurobehavioral deficits, traumatic brain injury

## Abstract

Interleukin-33 (IL-33) is a member of the interleukin-1 (IL-1) cytokine family and an extracellular ligand for the orphan IL-1 receptor ST2. Accumulated evidence shows that the IL-33/ST2 axis plays a crucial role in the pathogenesis of central nervous system (CNS) diseases and injury, including traumatic brain injury (TBI). However, the roles and molecular mechanisms of the IL-33/ST2 axis after TBI remain poorly understood. In this study, we investigated the role of IL-33/ST2 signaling in mouse TBI-induced brain edema and neurobehavioral deficits, and further exploited underlying mechanisms, using salubrinal (SAL), the endoplasmic reticulum (ER) stress inhibitor and anti-ST2L. The increase in IL-33 level and the decrease in ST2L level at injured cortex were first observed at 24 h post-TBI. By immunofluorescent double-labeled staining, IL-33 co-localized in GFAP-positive astrocytes, and Olig-2-positive oligodendrocytes, and predominantly presented in their nucleus. Additionally, TBI-induced brain water content, motor function outcome, and spatial learning and memory deficits were alleviated by IL-33 treatment. Moreover, IL-33 and SAL alone, or their combination prevented TBI-induced the increase of IL-1β and TNF-α levels, suppressed the up-regulation of ER stress, apoptosis and autophagy after TBI. However, anti-ST2L treatment could significantly invert the above effects of IL-33. Together, these data demonstrate that IL-33/ST2 signaling mitigates TBI-induced brain edema, motor function outcome, spatial learning and memory deficits, at least in part, by a mechanism involving suppressing autophagy, ER stress, apoptosis and neuroinflammation.

## Introduction

Traumatic brain injury (TBI), a major public health problem, continues to be a significant medical concern worldwide as the leading cause of morbidity and mortality in people under 40 years of age, with a significant burden on society (Jin et al., 2015). TBI also leads to the development of neurologic deficits, which is involved in a combination of effects in spatial learning, memory, cognition and personality (Santopietro et al., 2015). Although the medical technology has advanced significantly, the effective therapies for TBI are still limited. The hope for TBI treatment is based on the fact that much of the traumatic damage is caused by a secondary injury including biochemical and physiologic factors that are initiated by the primary mechanical TBI (Mustafa et al., 2010). Therefore, to study secondary injury composed of biochemical and physiologic factors might be an important aspect for TBI treatment.

Interleukin-33 (IL-33) is one of interleukin-1 (IL-1) family cytokines that are produced by many various categories of tissues including the central nervous system (CNS). It was also recognized as an extracellular ligand for ST2, the orphan IL-1 receptor (Schmitz et al., 2005). In recent years, IL-33 is increasingly reported to be involved in the pathogenesis of CNS diseases and injury, such as Alzheimer’s disease (AD; Xiong et al., 2014), multiple sclerosis (MS; Jafarzadeh et al., 2016), chronic pain (Longhi-Balbinot et al., 2016), intracerebral hemorrhage (ICH; Gao et al., 2017) and TBI (Wicher et al., 2017). Interestingly, a recent study demonstrated that levels of IL-33 expression in the brain was elevated from non-detectable levels, reaching a maximum after 72 h in both human TBI samples and mouse TBI model (Wicher et al., 2017). However, very little is known about the role and underlying intricate mechanisms of IL-33 in experimental TBI model.

In CNS, previous studies demonstrated that the production of IL-33 is primary derived from astrocytes and microglia, rather than neurons (Yasuoka et al., 2011; Gao et al., 2017). While most current findings indicate that IL-33 is expressed in neurons and oligodendrocytes, as well as in astrocytes and microglia (Gadani et al., 2015; Allan et al., 2016). The IL-33 receptor, ST2 (also named as T1, Der4 or IL-1RL1) was originally recognized in fibroblasts stimulated by oncogene or serum, which includes at least three isoforms by differential splicing such as a transmembrane receptor (ST2L), a soluble ST2 (sST2) and a variant ST2 (ST2V; Molofsky et al., 2015). Furthermore, the main source of ST2 is neurons, astrocytes and microglia (Allan et al., 2016). A recent study indicated that IL-33 plays a role in neuroinflammation with microglia/macrophages being cellular targets for this interleukin post-TBI (Wicher et al., 2017).

Autophagy, an evolutionarily conserved pathway, results in degradation of proteins and entire organelles in cells undergoing stress (Pozuelo-Rubio, 2011). Recent studies including ours have shown that autophagy is enhanced following TBI (Lai et al., 2008; Zhang and Ney, 2008; Luo et al., 2011), and inhibition of autophagy may attenuate TBI-induced traumatic damage and behavioral deficits (Luo et al., 2011). But the controversial roles and underlying mechanisms of autophagy after TBI still required to be further address in the future. It is now apparent that endoplasmic reticulum (ER) stress is also a potent trigger for autophagy, a self-degradative process that has an adaptive function (Lee et al., 2015). The ER stress response constitutes a cellular process that is triggered by various conditions that disturb folding of proteins in the ER (Lee et al., 2015). Numerous evidence indicates ER stress-induced cellular dysfunction even death as leading contributors to many various diseases and injury, including TBI (Harvey et al., 2015). As a specific inhibitor of eukaryotic translation initiation factor 2 subunit α (eIF2α) phosphatase enzymes, salubrinal (SAL) was used to investigate ER-mediated apoptosis after TBI in this study.

All above, we hypothesize that IL-33 as an important inflammatory regulator, may alleviate TBI-induced brain edema and neurobehavioral deficits. To investigate IL-33’s roles and underlying mechanisms undergoing by which IL-33 regulates autophagy, ER stress, apoptosis and neuroinflammation, several novel agents including IL-33, anti-ST2L and SAL were used in our mouse TBI model.

## Materials and Methods

### Animals and the TBI Model

This study was carried out in compliance with the NIH Guide for the Care and Use of Laboratory Animals. The protocol was approved by the Institutional Animal Care and Use Committee at Soochow University and Wenzhuou Medical University. All adult male ICR mice (20–25 g) were purchased from the Animal Center of the Chinese Academy of Sciences, Shanghai, China. Attempts were made to reduce animal suffering and the number of animals used. The procedure of TBI model had been described in detail previously (Luo et al., 2010, 2011). Briefly, the animals were deeply anesthetized with chloral hydrate (4% solution) and mounted in a Kopf stereotaxic system (David Kopf Instruments, Tujunga, CA, USA). A midline incision was then performed to expose the skull using aseptic techniques, and craniotomy was performed by hand-held trephine (Luo et al., 2010). The animals were subjected to TBI in left part of the brain using a weight-drop device: a 40 g weight dropped from 20 cm onto a 4-mm-diameter footplate resting on the dura with a controlled depth of 1.0 mm, as described previously (Luo et al., 2011).

### Drug Administration and Experimental Design

In this study, two sets of healthy male ICR mice were used. The first set of 120 mice were randomly divided into five groups: Sham group, TBI + PBS group, TBI + IL-33 group, TBI + SAL group and TBI + IL-33 + SAL group. IL-33 (50, 80, and 100 ng/μl; rmIL-33, Peprotech) or an equivalent volume of sterile phosphate-buffered saline (PBS) was administered intracerebroventricularly (i.c.v.) 30 min before TBI. SAL (1 mg/kg, EMD Chemicals Inc., Gibbstown, NJ, USA; Sokka et al., 2007; Logsdon et al., 2014), or an equivalent volume of PBS was injected intraperitoneally into mice immediately after TBI. The second batch of 18 mice was randomly divided into three groups: Sham group, TBI + PBS and TBI + Anti-ST2L. Some mice were intraperitoneally injected with 5 ug/mouse of ST2L neutralizing antibody (anti-ST2L; R&D Systems, Minneapolis, MN, USA) or an equivalent volume of PBS 30 min before TBI. For the injection into the ipsilateral ventricle, a small burr hole was made in the parietal region (1.0 mm posterior and 1.0 mm lateral to the Bregma, 2.5 mm in depth). At 24 h after TBI, most of the mice were anesthetized with chloral hydrate and blood samples were collected by cardiac puncture. The brain tissues were then removed and stored for further analysis at −80°C.

### Western Blot Analysis

Protein levels of apoptotic and autophagy-related proteins in brains of TBI and Sham groups were detected by western blot analysis, using the standard methods we described previously (Luo et al., 2013; Gao et al., 2017). In brief, each sample, 20 mg of protein, was loaded on a 10% or 12% SDS-PAGE gel using a constant current and then transferred to PVDF membranes on a wet electrotransferring unit (Bio-Rad). Membranes were incubated with antibodies to IL-33 (1:200, R&D), ST2L (1:500, abcam), LC3B (1:3000, Abcam), Beclin-1 (1:1000, Bioworld), P62 (1:1000, Abcam), GRP78 (1:1000, Abcam), Cleaved-Caspase-3 (CC-3; 1:500, Bioworld), Bcl-2 (1:500, Abcam) and β-actin (1:10,000, Sigma). Then, the membranes were washed and incubated with secondary antibodies conjugated to horseradish peroxidase for 2 h at room temperature. Immunoreactive proteins were detected with the ECL chemiluminescence system (Beyotime Institute of Biotechnology) and were obtained by Chemiluminescence Gel Imager. Films were automatically saved and densitometric analysis of the bands was performed with ImageJ.

### Immunofluorescence

IL-33 IR cell types in brains of TBI and Sham groups were identified by standard immunofluorescent methods, as described previously (Lee et al., 2012; Gao et al., 2017). Briefly, brains were perfused pericardially with PBS followed by 4% paraformaldehyde and cut into 6-μm sections with a cryotome. The defrosted tissue sections were incubated with antibodies to IL-33 (1:100; R&D), GFAP (1:500; Abcam), Olig-2 (1:500; Millipore), then the sections were incubated for 2 h at 4°C with an appropriate fluorescence-conjugated secondary antibody (1:200, Jackson Immuno-Research). The sections were stained for DAPI (1:5000, Beyotime Institute of Biotechnology) to visualize nucleus. Images were captured with a Fluorescence microscope (Zeiss).

### Motor Function and Morris Water Maze Testing

Motor Function Testing was assessed using a wire-grip test from day 1 to day 7 after TBI, according to the procedure described previously (Bermpohl et al., 2006). Briefly, animals were placed on a 45-cm-long metal wire suspended 45 cm above a foam pad, and the mice were allowed to traverse the wire for 60 s. The latency that a mouse remained on the wire within a 60 s interval was recorded, and wire-grip scores were quantitated using a five-point scale: zero point was given if mice were unable to remain on the wire for less than 30 s; one point was given if the mice failed to hold on to the wire with both sets of forepaws and hind paws together; two points were given if the animals held on to the wire with both forepaws and hind paws but not the tail; three points were given if the mouse used its tail along with both forepaws and both hind paws; four points were given if the mice moved along the wire on all four paws plus tail; and five points were given if the mice ambulated down one of the posts used to support the wire. The wire-grip test was performed in triplicate and an average value calculated for each mouse on each day of testing.

Morris water maze (MWM) test was applied to evaluate spatial learning and memory after TBI as described previously (Bermpohl et al., 2006; Mannix et al., 2011). Briefly, for the place navigation test, each time, the mice were placed in the water facing the wall in one of four starting locations (north east, south west, east, south) and allows up to 90 s to find and climb onto the submersed platform. Once the mice found the submersed platform within this time frame, they would be permitted to remain on the platform for an additional 10 s. However, the mice which failed to reach the submersed platform within the given time frame would be lifted onto the platform by the operator for 10 s to allow the animal to form memory of its location. The mice were then warmed under a heating lamp until the next trial. The escape latency was automatically recorded by a video/compute system. For the spatial probe test, the submersed platform was removed from the pool on day 15. Then each mouse was allowed to swim freely for 90 s and the frequency of passing through the target quadrant was recorded by a video tracking system.

### Brain Water Content

Brain edema was evaluated by a drying method at 24 h after TBI, as previously reported (Bermpohl et al., 2006; Gao et al., 2017). Briefly, the mice were anesthetized by intraperitoneal injection at the corresponding time points, the skull was stripped immediately after decapitation and the whole brain was removed and then removed the olfactory bulb and cerebellum. Subsequently, both hemispheres were separated along the anatomic midline, and the wet weight of each hemisphere was evaluated. The tissues were completely dried in an oven at 100°C for 48 h, and the dry weight of each hemisphere was recorded. The percentage water content (% water) was calculated according to the Elliott formula for each hemisphere: water content in brain tissue (%) = (wet weight − dry weight)/wet weight × 100%.

### Cytokine Enzyme-Linked Immune-Sorbent Assay (ELISA)

Interleukin-1β (IL-1β) and tumor necrosis factor-α (TNF-α) levels in serum were analyzed by Enzyme-Linked Immune-Sorbent Assay (ELISA; R&D), according to the manufacturers’ instructions. In brief, reagents, samples, and standard dilutions were prepared. Added 100 μL of Standard, Control, or sample per assigned well. Mixed by gently tapping the plate frame for 1 min and incubated for 1 h at 37°C. Aspirated each well and washed, repeating the process four times for a total of five washes. Then added 100 μL of Mouse IL-1β or TNF-α conjugate to each well and incubated for 1 h at 37°C. After washing, added 100 μL of Substrate Solution to each well with 20 min of incubation in the dark, the reaction was stopped by adding 100 μL of stop solution. Absorbance was read at 450 nm. Determinations were performed in triple, and results were expressed as mean OD ± SEM.

### Statistical Analysis

All the experiments were randomized and performed in a blinded manner. The behavioral data (motor function testing and escape latency of spatial probe test) was performed using two-way analysis of variance (ANOVA) for repeated measures. For the comparison of the frequency of passing through the platform quadrant of spatial probe test, one-way ANOVA with a Bonferroni test was used. The analysis of ELISA and Western blot were performed with one-way ANOVA analysis followed by *post hoc* Tukey’s test and Dunnett *t*-test for multiple comparisons, respectively. All data are based on at least three independent experiments. Results were presented as means ± standard error of the mean (SEM). A *p*-value less than 0.05 was considered significant. All statistical analyses were performed using the GraphPad Prism software suit.

## Results

### The Changes of IL-33 and ST2L Expression After TBI

**Figure 1:**
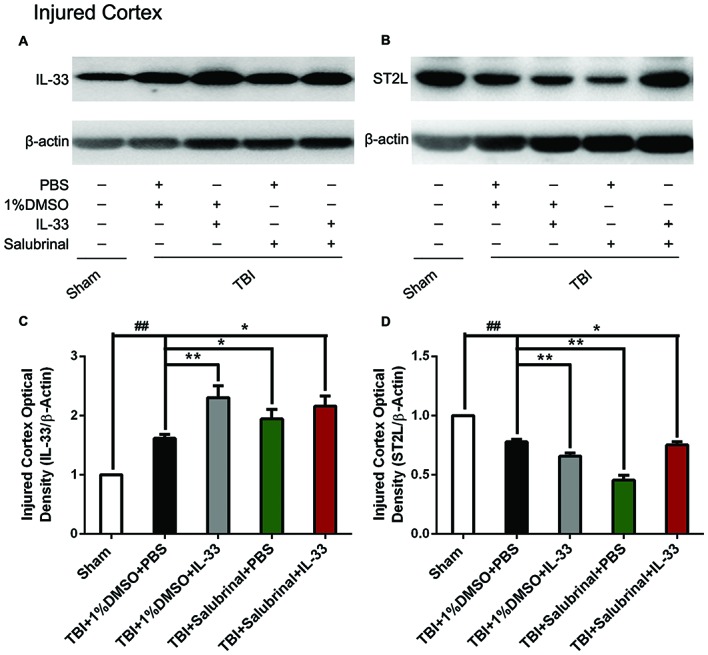
The changes of IL-33 and ST2L expression after traumatic brain injury (TBI). **(A,B)** The changes of IL-33 and ST2L expression in cerebral cortex after TBI. **(C,D)** Optical densities of the protein bands were quantitatively analyzed. IL-33 (*F*_(4,25)_ = 31.18; ^##^*P* < 0.01 vs. Sham group; **P < 0.01 vs. PBS group, *P < 0.05 vs. PBS group); ST2L (*F*_(4,25)_ = 56.55; ^##^*P* < 0.01 vs. Sham group; **P < 0.01 vs. PBS group, *P < 0.05 vs. PBS group). The data were expressed as means ± SEM (*n* = 6/group). Experiments are representative of three independent experiments.

To investigate whether IL-33 plays an important role in TBI, we detected the expression of IL-33 and its specific receptor ST2L at 24 h post-TBI (Figure 1). An increase in the levels of IL-33 expression was observed in TBI group (*P* < 0.01). The up-regulation of IL-33 expression was further enhanced by exogenous IL-33 injection alone (*P* < 0.01). By using SAL, the ER stress inhibitor, we found SAL alone or combined with IL-33 could up-regulate the IL-33 level, compared with TBI group (*P* < 0.05). Whereas, the level in the combined group has no significant difference compared to IL-33 or SAL group (*P* > 0.05).

Regarding the expression changes of the receptor ST2L of IL-33, we found that TBI obviously induced a decrease in ST2L expression, compared with Sham group (*P* < 0.01, Figure 1B). Compared with TBI group, the expression of ST2L protein was significantly further down-regulated by exogenous IL-33 or SAL alone (*P* < 0.01). However, the combined administration of SAL and IL-33 increased the level of ST2L expression, compared to IL-33 or SAL alone (*P* < 0.05).

### The Expression and Co-localization of IL-33 in Cerebral Tissues and Cells

**Figure 2:**
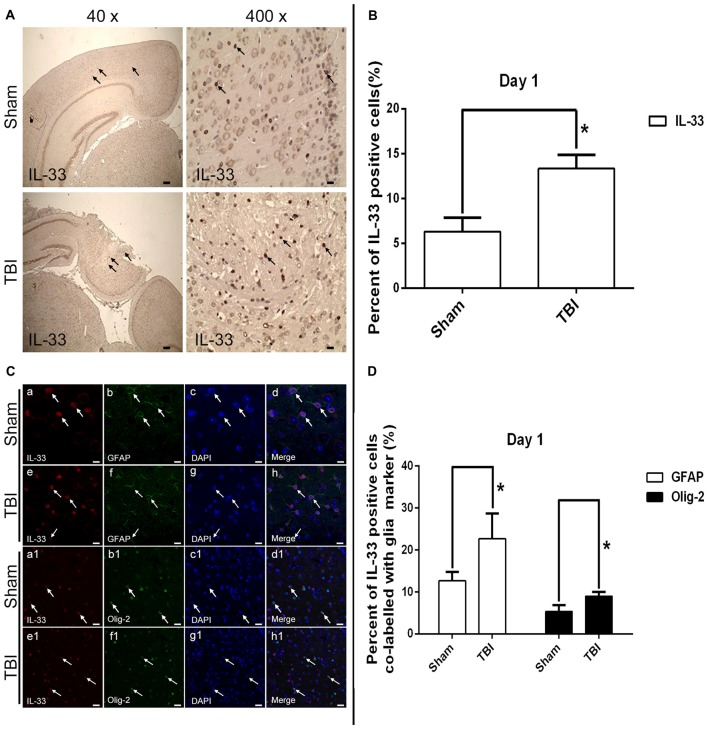
Expression of IL-33 and ST2 in cerebral cells and tissues. **(A)** Representative images showing IL-33 immunohistochemical stains of regions of interest surrounding the ipsilateral cortex by immunohistochemical staining. The injury site was zoomed in 40 and 400 times to illustrate the sample details. **(B)** Semi-quantitative analysis of IL-33 positive cells relative to the total number of cells. **(C)** Examples of double labeling were indicated with white arrows after TBI. **(a–h)** Co-localization of IL-33-like immnoreactivity and GFAP. Bar 50 μm. **(a_1_–h_1_)** Co-localization of IL-33-like immnoreactivity and Olig-2. Bar 50 μm. **(D)** Semi-quantitative analysis of glia type-cell contributions to the IL-33-positive cell population. The data were expressed as means ± SEM (*n* = 6/group). *P < 0.05 vs. Sham group. Experiments are representative of three independent experiments.

By utilizing immunohistochemical staining, we next examined the expression and localization of IL-33 in cells (Figures 2A,B). The results showed that TBI significantly resulted in an increase in IL-33 expression, and IL-33 staining was localized primarily in the nucleus (*P* < 0.05).

To further demonstrate the phenotype of the IL-33 immune-reactive (IR) cells after TBI, we detected co-localization of IL-33 with specific markers for astrocytes (GFAP; Figures 2Ca–h) and oligodendrocytes (Olig-2; Figures 2Ca1–h1), respectively. IL-33 co-localized in GFAP-positive astrocytes and predominantly presented in its nucleus. In parallel, IL-33 also co-expressed in Olig-2-positive oligodendrocytes. Moreover, quantitative analysis of the two types of double-labeled cells mentioned above were obviously up-regulated in TBI group, compared with Sham group (*P* < 0.05, Figure 2D).

### IL-33 Alleviates TBI-Induced Brain Edema, Motor Function Deficits and Performance in Morris Water Maze (MWM) Test

**Figure 3:**
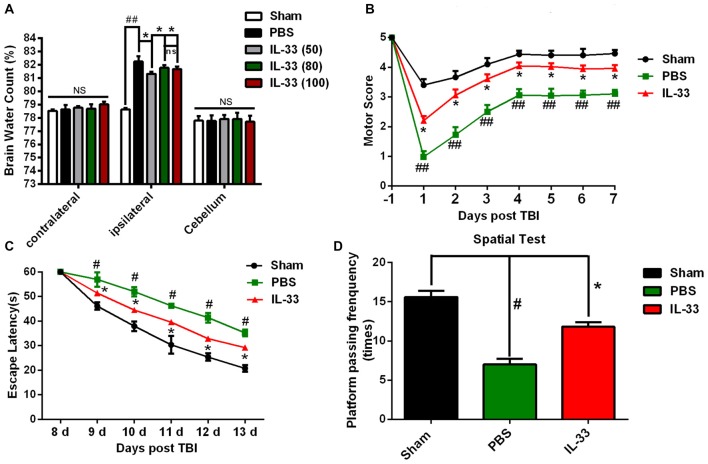
IL-33 alleviated TBI-induced brain edema, improved TBI-induced motor deficits and performance in Morris water maze (MWM) test. **(A)** IL-33 pretreatment significantly reduced cerebral edema at different concentrations in the range of 50–100 ng/μl in the ipsilateral cortex, and the brain water content was measured at 24 h post-TBI (*F*_(4,25)_ = 50.56; ^##^*P* < 0.01 vs. Sham group; *P < 0.05 vs. PBS group). No significance (NS) was observed among all groups, both in the contralateral hemisphere and cerebellum. **(B)** IL-33 pretreatment accelerated the recovery of TBI-induced motor function deficits (*F*_(14,96)_ = 22.94; ^##^*P* < 0.01 vs. Sham group; *P < 0.05 vs. PBS group). **(C)** In MWM test, mean escape latency for each group was plotted during day8-day13 (*F*_(10,72)_ = 10.18; ^#^*P* < 0.05 vs. Sham group; *P < 0.05 vs. PBS group). **(D)** The frequencies of crossing the platform were recorded on day 15 (*F*_(2,15)_ = 35.49; ^#^*P* < 0.05 vs. Sham group; *P < 0.05 vs. PBS group). The data were expressed as means ± SEM (*n* = 6/group). Experiments are representative of three independent experiments.

TBI led to a significant increase in the percentage of water content in the injured ipsilateral cortex at 24 h post-TBI, compared with Sham group (*P* < 0.05, Figure 3A). IL-33 (50, 80, and 100 ng/μl) treatment obviously reduced the percentage of water content in the injured hemisphere (*P* < 0.05). The optimum concentration of IL-33 for the best therapeutic effect was 50 ng/μl (*P* < 0.05). However, there were no significant differences among all groups, both in the contralateral hemisphere and cerebellum (*P* > 0.05).

In addition to reducing brain edema, IL-33 pretreatment remarkably improved the recovery of motor functional outcome on days 1–7 post TBI, compared with TBI group (*P* < 0.05, Figure 3B). The escape latencies of MWM on days 8–13 were significantly longer in TBI group than that in Sham group (*P* < 0.05), but the latencies were significantly reduced in IL-33 pretreatment group, compared with TBI group (*P* < 0.05, Figure 3C). For the spatial probe test, TBI induced a significant decrease in the frequency of passing through the platform quadrant, compared with Sham group (*P* < 0.05). However, IL-33 pretreatment significantly inverted the reduction in the probe tests, compared with TBI group (*P* < 0.05, Figure 3D). The above results implied that exogenous administration of IL-33 might be beneficial to motor function, learning and memory recovery after TBI in mice.

### IL-33 Acts as an Inflammatory Regulator in TBI

**Figure 4:**
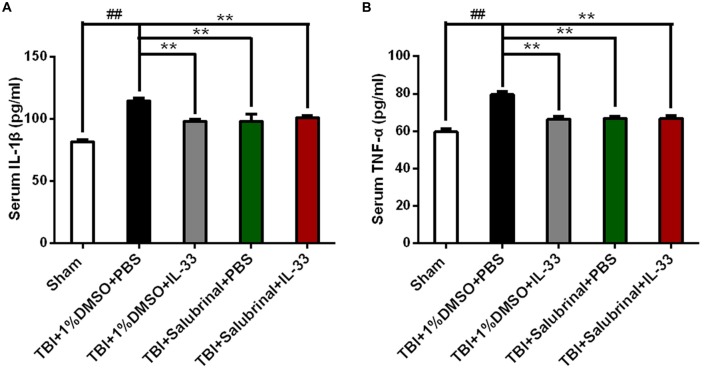
IL-33 is an anti-inflammation cytokine after TBI. **(A,B)** The expression levels of Interleukin-1β (IL-1β) and TNF-α were measured by enzyme-linked immune-sorbent assay (ELISA). TBI-induced TNF-α and IL-1β expression increase was inverted by IL-33 pretreatment in serum after TBI. IL-1β (*F*_(4,25)_ = 16.09; ^##^*P* < 0.01 vs. Sham group; **P < 0.01 vs. PBS group); TNF-α (*F*_(4,25)_ = 21.89; ^##^*P* < 0.01 vs. Sham group; **P < 0.01 vs. PBS group). The data were expressed as means ± SEM (*n* = 6/group). Experiments are representative of three independent experiments.

To investigate whether IL-33 affects TBI-induced inflammatory response, ELISA was carried out to assess the levels of inflammatory cytokines. The results showed that TBI significantly induced the up-regulation of IL-1β (*P* < 0.01, Figure 4A) and TNF-α (*P* < 0.01, Figure 4B) expression in the serum of mice with TBI. However, IL-33 pretreatment significantly inhibited the up-regulation of IL-1β and TNF-α levels (*P* < 0.01). Furthermore, SAL alone or combined with IL-33 could also down-regulated the expression of the above mentioned two types of cytokines, compared with TBI group (*P* < 0.01).

### IL-33/ST2 Signaling Pathway Inhibits Endoplasmic Reticulum Stress (ERS) After TBI

**Figure 5:**
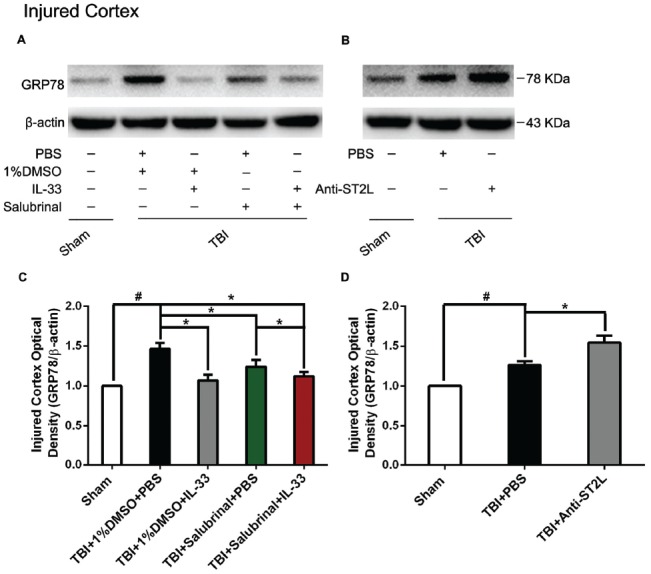
IL-33/ST2L signaling significantly down-regulated the level of endoplasmic reticulum stress (ERS) related-protein GRP78 after TBI. **(A)** IL-33 pretreatment significantly suppressed GRP78 expression (*F*_(4,25)_ = 7.669; ^#^*P* < 0.05 vs. Sham group; *P < 0.05 vs. PBS group). **(B)** Anti-ST2L pretreatment significantly promoted GRP78 expression (*F*_(2,15)_ = 34.60; ^#^*P* < 0.05 vs. Sham group; *P < 0.05 vs. PBS group). **(C,D)** Optical densities of the protein bands were quantitatively analyzed, and normalized with loading control β-actin. The data were expressed as means ± SEM (*n* = 6/group). Experiments are representative of three independent experiments.

To investigate the role of IL-33/ST2L signaling pathway in endoplasmic reticulum stress (ERS) after TBI, western blotting was performed to detect the expression levels of ER stress-related protein (GRP78). As shown in Figure 5, TBI significantly led to the increase of GRP78 expression, compared with Sham group (*P* < 0.05). By contrast, IL-33 and SAL alone, or their combination significantly inhibited the up-regulation of GRP78 expression, compared with TBI group (*P* < 0.05). Moreover, their combination could also remarkably reduce GRP78 expression, compared with SAL alone (*P* < 0.05).

To further explore whether ST2L signaling pathway is involved in IL-33’s anti-ERS effects, anti-ST2L was intraperitoneally injected into mice suffering from TBI. The results indicated that anti-ST2L treatment significantly increased GRP78 expression after TBI (*P* < 0.05, Figure 5B). The above results indicate that IL-33 exerts an inhibitory effect on ER stress response through the ST2L signaling pathway after TBI.

### IL-33/ST2L Signaling Pathway Is Involved in Apoptosis Activation After TBI

**Figure 6:**
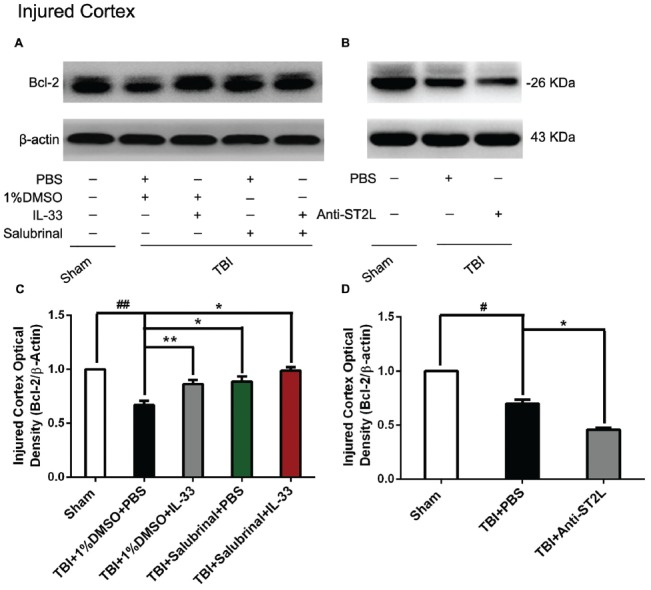
IL-33/ST2L signaling inverted TBI-induced Bcl-2 decrease. **(A)** IL-33 pretreatment inhibited TBI-induced down-regulation of Bcl-2 expression in cortex after TBI (*F*_(4,25)_ = 38.17; ^##^*P* < 0.01 vs. Sham group; **P < 0.01 vs. PBS group, *P < 0.05 vs. PBS group). **(B)** Anti-ST2L pretreatment obviously inhibited Bcl-2 expression in cortex tissues after TBI (*F*_(2,15)_ = 94.93; ^#^*P* < 0.05 vs. Sham group; *P < 0.05 vs. PBS group). **(C,D)** Optical densities of the protein bands were quantitatively analyzed, and normalized with loading control β-actin. The data were expressed as means ± SEM (*n* = 6/group). Experiments are representative of three independent experiments.

**Figure 7:**
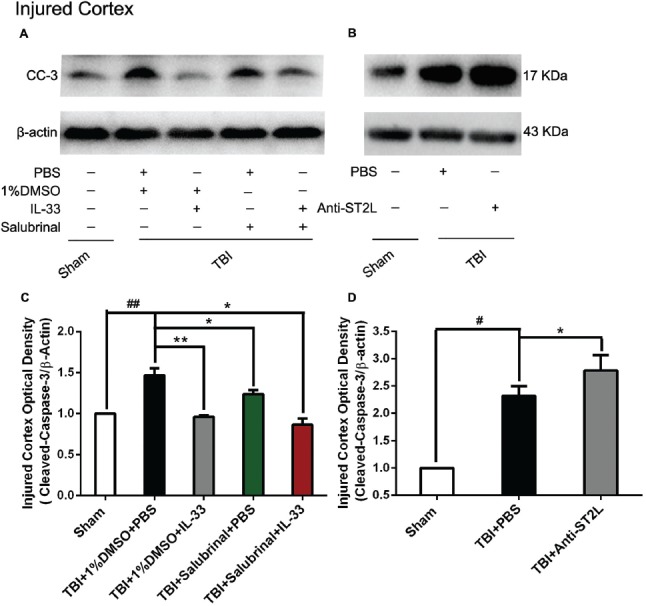
IL-33/ST2L signaling inverted TBI-induced Cleaved-Caspase-3 (CC-3) increase. **(A)** IL-33 pretreatment significantly down-regulated CC-3 expression in cortex after TBI (*F*_(4,25)_ = 38.17; ^##^*P* < 0.01 vs. Sham group; **P < 0.01 vs. PBS group, *P < 0.05 vs. PBS group). **(B)** Anti-ST2L pretreatment obviously up-regulated CC-3 expression in ipsilateralcortex after TBI (*F*_(2,15)_ = 40.57; ^#^*P* < 0.05 vs. Sham group; *P < 0.05 vs. PBS group). **(C,D)** Optical densities of the protein bands were quantitatively analyzed, and normalized with loading control β-actin. The data were expressed as means ± SEM (*n* = 6/group). Experiments are representative of three independent experiments.

To explore whether IL-33/ST2L signaling plays a key role in apoptosis after TBI, we used exogenous administration of IL-33 and Anti-ST2L to observe the changes of apoptosis-related proteins, such as Bcl-2 and CC-3. The results revealed that TBI induced a significant decrease in Bcl-2 expression (*P* < 0.01, Figures 6A,C) and an increase in CC-3 expression (*P* < 0.01, Figures 7A,C) in cerebral cortex at 24 h post-TBI (*P* < 0.01). Whereas exogenous injection with IL-33 and SAL alone, or their combination significantly up-regulated the level of Bcl-2 expression and down-regulated the level of CC-3 expression (*P* < 0.01; *P* < 0.05), suggesting that IL-33 exerts anti-apoptotic effects by enhancing Bcl-2 and decreasing CC-3 expression following TBI.

To confirm whether IL-33 plays an anti-apoptotic effect on TBI through ST2L signaling pathway. By using intraperitoneal injection of Anti-ST2L, we found that Anti-ST2L treatment significantly down-regulated the expression level of Bcl-2 (*P* < 0.05, Figures 6B,D) and up-regulated the expression level of CC-3 (*P* < 0.05, Figures 7B,D) at 24 h post-TBI, thus reversely demonstrating the anti-apoptotic effect of IL-33 on TBI, and this effect was mediated by the ST2L signaling pathway.

### IL-33/ST2L Signaling Pathway Is Involved in Autophagic Activation After TBI

**Figure 8:**
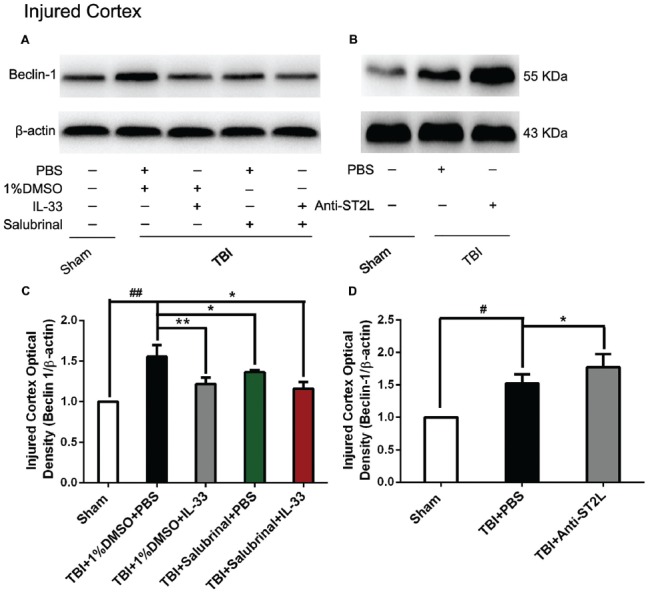
IL-33/ST2L signaling suppressed Beclin-1 expression after TBI. **(A)** IL-33 pretreatment significantly reduced Beclin-1 expression (*F*_(4,25)_ = 12.06; ^##^*P* < 0.01 vs. Sham group; **P < 0.01 vs. PBS group, *P < 0.05 vs. PBS group). **(B)** Anti-ST2L pretreatment significantly increased Beclin-1 expression (*F*_(2,15)_ = 14.03; ^#^*P* < 0.05 vs. Sham group; *P < 0.05 vs. PBS group). **(C,D)** Optical densities of the protein bands were quantitatively analyzed, and normalized with loading control β-actin. The data were expressed as means ± SEM (*n* = 6/group). Experiments are representative of three independent experiments.

**Figure 9:**
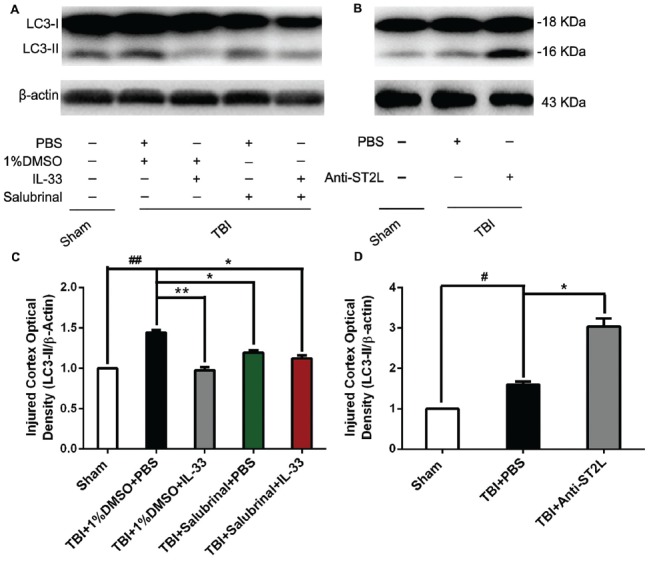
IL-33/ST2L signaling significantly reduced the level of LC3-II expression after TBI. **(A)** IL-33 pretreatment significantly reduced the LC3-II/β-actin ratio (*F*_(4,25)_ = 151.9; ^##^*P* < 0.01 vs. Sham group; **P < 0.01 vs. PBS group, *P < 0.05 vs. PBS group). **(B)** Anti-ST2L pretreatment significantly increased the LC3-II/LC3-I ratio (*F*_(2,15)_ = 82.28; ^#^*P* < 0.05 vs. Sham group; *P < 0.05 vs. PBS group). **(C,D)** Optical densities of the protein bands were quantitatively analyzed, and normalized with loading control β-actin. The data were expressed as means ± SEM (*n* = 6/group). Experiments are representative of three independent experiments.

**Figure 10:**
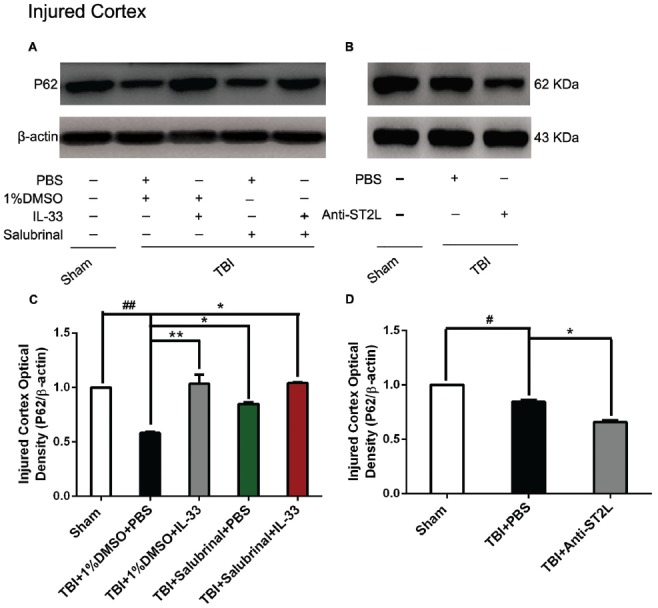
IL-33/ST2L signaling significantly increased P62 expression after TBI. **(A)** IL-33 pretreatment significantly up-regulated P62 expression (*F*_(4,25)_ = 21.57; ^##^*P* < 0.01 vs. Sham group; **P < 0.01 vs. PBS group, *P < 0.05 vs. PBS group). **(B)** Anti-ST2L pretreatment significantly down-regulated P62 expression (*F*_(2,15)_ = 122.3; ^#^*P* < 0.05 vs. Sham group; *P < 0.05 vs. PBS group). **(C,D)** Optical densities of the protein bands were quantitatively analyzed, and normalized with loading control β-actin. The data were expressed as means ± SEM (*n* = 6/group). Experiments are representative of three independent experiments.

**Figure 11:**
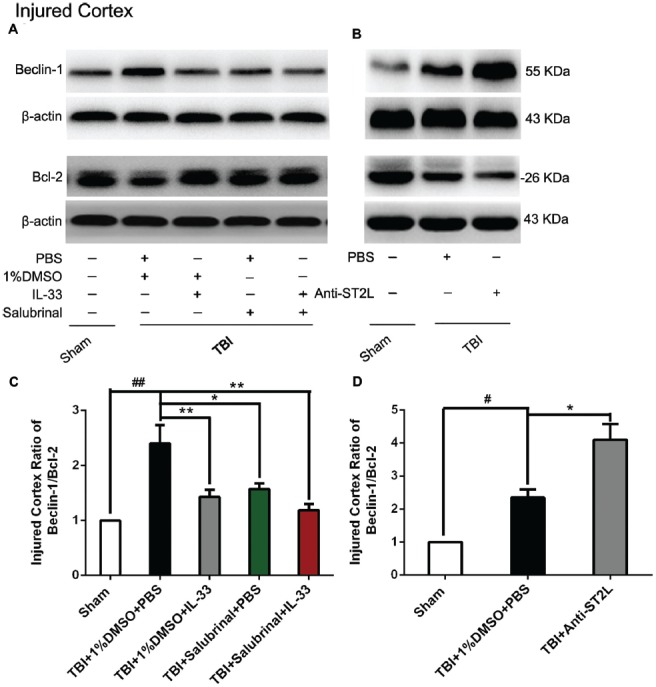
IL-33/ST2L signaling significantly down-regulated the Beclin-1/Bcl-2 ratio after TBI. **(A)** IL-33 pretreatment down-regulated the Beclin-1/Bcl-2 ratio after TBI (*F*_(4,25)_ = 16.83; ^##^*P* < 0.01 vs. Sham group; **P < 0.01 vs. PBS group, *P < 0.05 vs. PBS group). **(B)** Anti-ST2L pretreatment significantly up-regulated the Beclin-1/Bcl-2 ratio (*F*_(2,15)_ = 40.62; ^#^*P* < 0.05 vs. Sham group; *P < 0.05 vs. PBS group). **(C,D)** Optical densities of the protein bands were quantitatively analyzed, and normalized with loading control β-actin. The data were expressed as means ± SEM (*n* = 6/group). Experiments are representative of three independent experiments.

To confirm the effect of IL-33 on autophagic activation after TBI, western blot analysis was performed to assess the expression of autophagy-associated proteins such as, Beclin-1 (Figure 8), LC3-II (Figure 9), and p62 (Figure 10). We found that a dramatic elevation in Beclin-1 (*P* < 0.01, Figure 8A) and LC3-II (*P* < 0.01, Figure 9A) levels, and a significant down-regulation of p62 expression level (*P* < 0.01, Figure 10A) were observed in TBI group at 24 h post-TBI. However, IL-33 and SAL alone, or their combination markedly inverted TBI-induced expression changes in the three types of proteins mentioned above (*P* < 0.01; *P* < 0.05). Moreover, they also significantly down-regulated the Beclin-1/Bcl-2 ratio after TBI (*P* < 0.01, Figures 11A,C).

To further explore whether ST2L was involved in the mechanism of IL-33 in inhibiting autophagy after TBI, antibodies to ST2L were intraperitoneally injected into mice suffering from TBI. We found that administration of anti-ST2L increased Beclin-1 (*P* < 0.05, Figure 8B) and LC3-II (*P* < 0.05, Figure 9B) expression, and decreased P62 expression (*P* < 0.05, Figure 10B), as well as down-regulated the Beclin-1/Bcl-2 ratio after TBI (*P* < 0.01, Figures 11B,D). The above mentioned data demonstrate that the anti-autophagic role of IL-33 in TBI was mediated by ST2L signaling pathway.

## Discussion

The neuroprotective effects of IL-33, an important inflammatory regulator, have been demonstrated in previous studies involving CNS diseases and injury, such as AD (Xiong et al., 2014), MS (Jafarzadeh et al., 2016), chronic pain (Longhi-Balbinot et al., 2016), ICH (Gao et al., 2017); however, the roles of IL-33 in TBI and the underlying mechanisms needed to address. Thus, the current study was aimed to investigate the neuroprotective effects of IL-33 on mice subjected to TBI. The major findings of this manuscript include: (1) IL-33 treatment inhibited autophagy, ER stress, apoptosis, neuroinflammation and improved neurologic outcomes after TBI. (2) The combination of IL-33 and SAL did not achieve better results than IL-33 or SAL alone. (3) ST2L neutralizing antibody treatment inverted the neuroprotective effects of IL-33. The above results indicated that IL-33 provided neuroprotective role in TBI, at least in part, through ST2L signaling.

To date, the role of IL-33 in TBI model remains controversial. The formation of cerebral edema is the most important pathological response following TBI. Emergence of acute edema, rapid progress, can cause increased intracranial pressure, mild cause neurological damage, but severe cases can cause the form of hernia or even life-threatening. TBI-induced inflammatory response, cell death and hypoxia and other factors can lead to cerebral edema or accompanied by the formation of cerebral edema, which can result in high mortality and disability (Chen et al., 2007; Gu et al., 2016). Blixt et al. (2018) found that multiple molecular mechanisms were involved in cerebral edema after TBI, including the rupture of small vessels, the disruption of Blood Brain Barrier (BBB) and activation of inflammation response. The BBB was disrupted following TBI, and the resulting cerebral edema led to the elevation in intracranial pressure and thus resulted in poor outcomes (Blixt et al., 2018). To improve neurologic outcomes following TBI, pharmacological therapies are designed to slow the development of cerebral edema and to attenuate the BBB disruption. We observed that administration of IL-33 with dosages of 50 ng/μl, 80 ng/μl or 100 ng/μl could significantly alleviate TBI-induced brain edema, and the optimum concentration of IL-33 for the best therapeutic effect is 50 ng/μl. Furthermore, we further investigate the impact of IL-33 on motor function and learning and memory function after TBI. The present results revealed that IL-33 pretreatment improved TBI-induced motor deficits and resulted in a significant amelioration in injured animals in hidden platform and spatial learning probe trials, suggesting the neuroprotective effect of IL-33 on TBI-induced motor function outcomes, spatial learning and memory deficits.

Notably, previous studies suggested that IL-33 functioned as a bifunctional role in CNS diseases and might be associated with its expression, synthesis and secretion (Miller et al., 2008; Moussion et al., 2008). Numerous literatures identified that IL-33 is dominantly produced by endothelial cells and astrocytes but not by microglia or neurons *in vitro* cellar culture (Manetti et al., 2010; Yasuoka et al., 2011). Other reports revealed that IL-33 was mainly localized in astrocytes, microglia and neurons after brain injury *in vivo* (Huang et al., 2015; Gao et al., 2017). We speculate the reason for the discrepancy may be caused by the different experiment settings, environments between *in vitro* and *in vivo*, or because of tissue repair process after acute brain injury. Consistent with previous reports, our current results demonstrated that TBI led to a significant up-regulation in the level of IL-33 expression. Moreover, IL-33 was either localized in the cytoplasm or nucleus of cerebral cells by immunohistochemical staining, and it almost exclusively located in the nucleus of astrocytes, and in the cytoplasm of oligodendrocytes. We suspected that glial-derived IL-33 might play a role in rescuing and protecting neurons against TBI through ST2L receptor.

The pathological changes involved in TBI mainly include oxidative stress, Ca^2+^ homeostasis disruptions, ER stress, autophagic dysfunction, excitotoxicity and free-radical generation, all of which could lead to apoptosis and neuroinflammation (Pearn et al., 2017). Schiavone et al. (2017) investigated the possible contribution of NOX2-derived oxidative stress to neuropathological alterations associated to TBI on post mortem brain samples of subjects died following TBI, with a particular focus on the loss of parvalbumin (PV)-positive interneurons. By using delayed NOX inhibition or global genetic NOX2 knockout method, Wang et al. (2017) provided a direct link between NADPH oxidase 2 (NOX2) and the NF-κB pathway in microglia/macrophages after TBI, and exploited a novel mechanism by which NOX2 activation leaded to the enhanced inflammatory response and neuronal damage after brain injury. In addition, the combo therapy (NOX2 inhibitor apocynin + Nrf2 activator TBHQ) could improve motor and cognitive outcome and decrease cortical lesion volume compared with vehicle group, indicating that preventing ROS formation and potentiating ROS disposal concurrently are efficacious after TBI (Chandran et al., 2017).

During the conditions of nutrient limitation, the role of autophagy is to maintain cell viability via the bulk degradation of cytoplasmic material through generating amino acids and energy, (Messer, 2017). The presence of autophagy in dying cells has also been reported to be a stress response mechanism to prolong cell viability (Diskin et al., 2005; Erlich et al., 2006). However, recent studies report that autophagy is a process that can promote and affect programmed cell death (Erlich et al., 2007; Lin and Baehrecke, 2015). Autophagy has been reported to be associated with various brain diseases or injury, including neurodegenerative diseases (Rubinsztein et al., 2005), cerebral ischemia (Balduini et al., 2009) and TBI (Luo et al., 2011). It is still unclear whether autophagy functions as a cell death or a cell survival pathway (Shintani and Klionsky, 2004; Lin and Baehrecke, 2015). Recent studies including ours indicated that autophagy is increased following TBI or ICH (Lai et al., 2008; Zhang and Ney, 2008; Luo et al., 2011), and inhibition of autophagy provided neuroprotection in multiple experimental models of brain injury, including TBI (Zhang et al., 2014; Gao et al., 2017), and ICH (Gao et al., 2017). The above findings revealed that inhibition of autophagy might respect a novel and promising intervention for therapies of the nervous system diseases and injury including TBI. In this study, pretreatment with IL-33 significantly inhibited TBI-induced increase of LC3II and Beclin-1 expression, and maintained p62 levels after TBI, indicating IL-33 could inhibit TBI-induced autophagic activation. Furthermore, anti-ST2L treatment obviously inverted the role of IL-33 in inhibiting autophagy, suggesting IL-33 might suppress autophagic activity via the ST2L signaling pathway following TBI. Additional studies are required to determine the underlying mechanisms of IL-33 regulating autophagy following TBI.

ER stress is reported to a potent trigger for autophagy, a self-degradative process (Lee et al., 2015). ER stress is a hallmark of numerous common diseases and injuries in conditions where: (1) the stress is so strong and/or prolonged to the point that cells succumb to death; or (2) the ability to overcome ER stress is impaired by a pathological condition. The ER stress response constitutes a cellular process that is triggered by a variety of conditions that disturb folding of proteins in the ER (Lee et al., 2015). Numerous evidence indicates ER stress-induced cellular dysfunction even cell death as major contributors to various diseases and injury, including TBI (Harvey et al., 2015). However, few effective treatments for ER stress following TBI have been developed by preclinical and clinical studies (Begum et al., 2014; Lucke-Wold et al., 2015). Thus, the role and regulation of ER stress in TBI still require further investigation. By using SAL, a specific inhibitor of the ER stress, we found that SAL treatment remarkably up-regulated IL-33 level, inhibited inflammatory responses, ERS, apoptosis and autophagy after TBI. Additionally, pretreatment with IL-33 evidently inhibited TBI-inducted elevation of an ER stress marker, GRP78 level in the injured cortex post-TBI; however, no more significant inhibition of ER stress was not detected by the combination of IL-33 and SAL. Furthermore, anti-ST2L treatment obviously inverted the role of IL-33 in inhibiting autophagy, suggesting a role of IL-33 in inhibiting ER stress, at least in part, by the ST2L signaling pathway following TBI.

Apoptosis has also contributed to programmed cell death in TBI (Luo et al., 2011). Among the identified apoptotic genes, Bcl-2 and caspase-3 are widely recognized as the most important apoptotic regulators, and their relative levels determine the fate of neural cells. We found that IL-33 treatment led to the decrease of CC-3 protein level and the increase of Bcl-2 protein level in the injured cortex after TBI. Furthermore, anti-ST2L treatment obviously inverted the role of IL-33 in inhibiting apoptosis after TBI. These data indicated that IL-33 suppressed the activation of apoptosis at least in part by the ST2L signaling pathway following TBI. Overall, the neuroprotective effect of IL-33/ST2L signaling axis on TBI is via inhibiting autophagy, ER stress and apoptosis, and the relationships among them can be quite intricate: (1) The relationship between ER stress and apoptosis: both ER stress and mitochondrial dysfunction contribute to modulate apoptotic after CNS insults. A major consequence of p-PERK over-activation is the induction of its down-stream transcription factor ATF4 that increases the expression of BAX, PUMA and BIM (Nakka et al., 2016). ATF4 can induce CHOP which in turn induces many downstream genes resulting in apoptosis and autophagy (Nakka et al., 2016). (2) The relationship between ER stress and autophagy: ER stress induces the formation of autophagosome through IRE1-JNK signaling pathway (Ogata et al., 2006). Moreover, mutations in the PERK phosphorylation site of eIF2α prevents *Atg2* up-regulation and conversion of LC3 further supports the notion that PERK pathway is a mediator of autophagy (Adhami et al., 2007). It seems that ER stress-induced autophagy could be neuroprotective based on the observation that SAL, the ER stress inhibitor could inhibit autophagic activation and exert neuroprotection after ischemic preconditioning (Szydlowska and Tymianski, 2010). (3) The relationship between apoptosis and autophagy: it appears that Bcl-2 interacting domain of Beclin-1 serves as a important point of crosstalk between apoptosis and autophagy (Decuypere et al., 2012). Our previous study showed that autophagy inhibitors such as 3-MA and BFA could inhibit the Beclin-1/Bcl-2 ratio, and active-caspase-3 after TBI (Luo et al., 2011), indicating the inhibition of autophagy provides neuroprotective effects via suppressing apoptosis. Therefore, the molecular crosstalk between ER stress, autophagy and apoptosis represents a cycle that can be pharmacologically targeted to inhibit cell death following acute injuries to CNS, including TBI. In our study, IL-33/ST2L signaling is suppressing autophagy, ER stress, and apoptosis. But the underlying molecular mechanisms by which ER is selected as autophagic cargo, and the crosstalk between ER stress-induced autophagy and activation of apoptosis after TBI are required to address.

A variety of mechanisms are involved in cell death, including apoptosis, autophagy and necrosis. In particular, inflammatory cytokines play a very important role in the development, progression and prognosis of CNS diseases and injuries. They are proteins and small molecule polypeptides produced by immune cells, mainly including tumor necrosis factor (TNF-α) and interleukin (IL-1β), which can transmit information between cells and has immunomodulatory function (Greve and Zink, 2009). To a certain extent, inflammatory cytokines can play a protective role against brain tissue injury, but excessive inflammatory reaction will cause harm to the body (Morganti-Kossmann et al., 2001, 2007). IL-33, as a novel cytokine, plays a double-edged sword in diseases and injuries (Russo and McGavern, 2016). In the study, we found that following TBI, IL-33 significantly reduced the levels of two pro*-*inflammatory cytokines, IL-1β and TNF-α, thereby inhibiting the inflammatory response. Previous studies by Gao et al. (2017) showed that IL-33 attenuated cerebral edema, inhibited neuronal cell death, and improved neurological impairment by inhibiting the expression of proinflammatory cytokines IL-1β and TNF-α in a model of cerebral hemorrhage. In addition, SAL treatment also inhibited the levels of the above two cytokines after TBI, which is the same as the anti-inflammatory tendency of IL-33. The above data suggested that IL-33 may play a protective role via down-regulating the levels of pro-inflammatory cytokines IL-1β and TNF-α after TBI.

In conclusion, we demonstrated that pretreatment with IL-33 provides neuroprotection by improving neurological function and ameliorating cerebral edema in a mouse model of TBI. Moreover, administration of IL-33 inhibited autophagy, ER stress and apoptosis and neuroinflamation after TBI. In parallel, anti-ST2L treatment could significantly invert the above effects of IL-33. Treatment with the ER stress inhibitor SAL confirmed the underlying relationship among ER stress, autophagy, IL-33 and neuroinflamation. We suggest that IL-33 mediated the inhibition of autophagy and ER stress may be an effective therapeutic strategy for TBI.

## Author Contributions

YG, CL and LT performed the experiments and analyzed data and were responsible for the conception and design of the study and manuscript writing and contributed to manuscript preparation. YG, MZ, TW, ZW, CG and GY performed animal experiments. YF, LY, HW and CG helped to process data.

## Conflict of Interest Statement

The authors declare that the research was conducted in the absence of any commercial or financial relationships that could be construed as a potential conflict of interest. The reviewer ZW declared a shared affiliation, though no other collaboration, with several of the authors YG, YF, LY, GY to the handling Editor
